# Effect of NiTi Cutting Efficiency on Generating Intra-Canal Splitting Forces During Root Canal Treatment: An *In Vitro* Study

**DOI:** 10.1055/s-0045-1813652

**Published:** 2025-12-23

**Authors:** Jalal K. Al-Awqati, Anas Al-Jadaa, Abdul R. Md. Saleh, Esraa Jaber

**Affiliations:** 1 Department of Clinical Dental Sciences, College of Dentistry, Ajman University, Ajman, United Arab Emirates; 2 Center of Medical and Bio-Allied Health Sciences Research, Ajman University, Ajman, United Arab Emirates

**Keywords:** Bovine teeth, VRF, ICSF, cutting efficiency, wedging effect, root canal preparation, dentin, stress strain

## Abstract

**Objective:**

This study aims to measure the effect of the lateral cutting efficiency of reciprocating and rotary NiTi files on the produced intracanal splitting forces (ICSF) during root canal preparation.

**Materials and Methods:**

Forty-eight mandibular anterior bovine teeth with straight roots were used to create 48 simulated premolar roots and 26 enamel-dentin disks. The required sample size was determined through power analysis conducted with G ^*^ Power 3.1.9.7 software, utilizing data from preliminary studies. Based on a large effect size (0.8), significance level of 0.05, and statistical power of 80%, the analysis indicated a need for at least 21 specimens per group for splitting force evaluation and 10 specimens for cutting efficiency assessment. To maintain sufficient statistical power and accommodate possible sample attrition, the study employed 24 specimens per group for splitting force analysis and 13 specimens for cutting efficiency evaluation. The investigation examined two file systems with comparable heat treatment but distinct kinematics, geometries, and designs to assess the intracanal stress forces generated during preparation and their respective cutting abilities in dentin discs. The tested instruments were WaveOne-Gold (WOG) and Pro-Taper GOLD (PTG). Random allocation was performed using a computer-generated randomization sequence (Random.org). Specimens were numbered consecutively and assigned to groups using block randomization to ensure equal group sizes. Tests were carried out on a custom-made platform under automated conditions. The data collected by the force gauge is in newtons (N), and the cutting efficiency was calculated by measuring the depth of cut produced in dentin in mm. Data analysis was carried out with the Kolmogorov–Smirnov test and one-way Anova.

**Results:**

The splitting forces test was significantly higher in the PTG group (S1) file when compared to other PTG and WOG files. The force generated in WOG strokes presented an ascending manner as the file went deeper apically. The cutting efficiency of PTG (F2) was significantly higher than WOG's primary file. However, no significant correlation between splitting force and file cutting efficiency was detected.

**Conclusion:**

WOG single reciprocating file produced significantly lower splitting force values with significantly less aggressive dentin cutting compared to PTG multi-sequence rotary files. File design, kinematics, depth of strokes, and the maximum diameter of each file at the coronal third in relation to canal diameter may be influencing factors in generating splitting forces.

## Introduction

The structural integrity of human teeth faces challenges from both functional and iatrogenic stresses, potentially leading to cracks or fractures. These failures range from minor crown cracks to complete tooth splits during normal masticatory function. 1 Vertical root fracture (VRF) represents a particularly complex type, characterized by a fracture line that may originate apically and extend coronally, begin coronally and progress apically, or emerge from within the root itself. Fractures beginning in the apical third commonly relate to endodontic treatment, while those starting cervically typically stem from occlusal forces. 2

Epidemiological studies report VRF incidence rates of approximately 2.3%, 3 with higher prevalence among males over 40 who have undergone endodontic treatment. This suggests endodontically treated teeth may be predisposed to VRF due to weakened structural integrity, further compromised during therapy. 4 A longitudinal study by Sjögren et al revealed that among 68 extracted teeth with prior endodontic treatment, 30.8% exhibited VRF. 5

VRF frequency varies by tooth type and position. Testori et al found premolars showed higher VRF incidence following endodontic treatment, 6 while Chan et al identified first molars as most frequently affected. 7 Conversely, canines demonstrated the lowest susceptibility. 4 Clinically, VRF poses substantial challenges, often leading to progressive bone resorption and periodontal tissue deterioration. Although various treatments exist, reliable solutions remain limited to surgical resection of the fractured root or complete extraction, 8 emphasizing the importance of preventative strategies based on understanding etiological factors. 1

One significant factor involves root canal wall damage from chemo-mechanical procedures, resulting in microcracks and craze lines that potentially precede VRF. 9 10 11 These defects may worsen under occlusal forces or subsequent restorative treatments, eventually progressing to full fractures. 3 12 While adequate enlargement is essential for microbial control, over-instrumentation weakens root structure and increases VRF risk. Simultaneously, proper stress management during preparation and obturation is crucial to prevent microstructural damage. 13

As Biomechanical shaping procedures correlate with defect development, 14 multiple factors are suggested to influence this process, including instrument taper, size, cross-sectional design, materials, manufacturing techniques, and mechanical preparation approaches. 15

Nevertheless, Ahn et al's systematic review found no definitive causal relationship between instrument kinematics and dentinal defect creation. Reciprocating motion potentially offers mechanical advantages by periodically disengaging from dentine, imposing less torsional stress on canal walls 16 and limiting possible crack initiation risk. 15

The impact of preparation steps continues to be debated. While some studies report that single reciprocating file systems cause more dentinal defects than stepwise sequences in rotary systems, other studies have found no significant differences between these approaches. 17 18 Conversely, some research indicates full-sequence rotary systems generate more defects than single-file reciprocating systems. 19 20

Despite concerns, some investigations suggest canal preparation can proceed without inducing dentinal defects, with contradictory outcomes potentially stemming from methodological variations. An *in vivo* Micro-CT study of premolars following clinical preparation reported no observable crack initiation, 21 supported by several *in vitro* studies. 22 23 24 Recent Micro-CT analysis revealed microcracks after instrumentation mirrored those present beforehand, suggesting preparation may not create new microcracks. 25

Understanding dentinal cracks is crucial given their potential to compromise outcomes. A prospective periapical surgery study demonstrated significantly lower healing rates in teeth exhibiting dentinal cracks. However, whether all microcracks eventually develop into VRFs remains uncertain, leaving long-term clinical implications open to interpretation. 26

To prevent VRFs, Rivera et al advised minimizing intra-radicular dentine removal and avoiding internal wedging forces during treatment. These forces arise from vertical pressure exerted by endodontic files, creating hydrostatic pressure capable of splitting canal walls. 1 When acting on opposing canal areas, these forces generate horizontal stresses compromising root structural integrity. Research examining wedging forces induced by tapered rotary instruments has utilized adapted testing platforms to measure resulting horizontal splitting forces, indicating previous assessments may have underestimated their severity. 27 28

The relationship between root canal preparation and structural crack development, potentially leading to VRFs, remains complex and underinvestigated. Microcracks represent potential VRF precursors. While chemo-mechanical preparation has frequently been implicated in canal wall damage, the precise roles of different instrumentation techniques, file geometries, and motion kinematics continue to be explored. 9 10 11

Advancing understanding of these factors is essential for refining root canal techniques and improving instrument safety. This investigation aims to test the lateral cutting efficiency effect of two NiTi file motions (ProTaper-Gold [PTG] and WaveOne-Gold [WOG]) on the produced intracanal splitting forces (ICSF) during root canal preparation. Despite extensive research on these file systems, the direct relationship between their cutting efficiency and ICSF remains underexplored. Therefore, the aims of this study were to compare the ICSF generated by WOG reciprocating and PTG rotary file systems. In addition, it is aimed to evaluate the effect of the lateral cutting efficiency of both systems and its relation to the generated ICSF. This insight is expected to help in file selection for minimizing root fracture risk during endodontic treatment.

## Materials and Methods

**Fig. 1:**
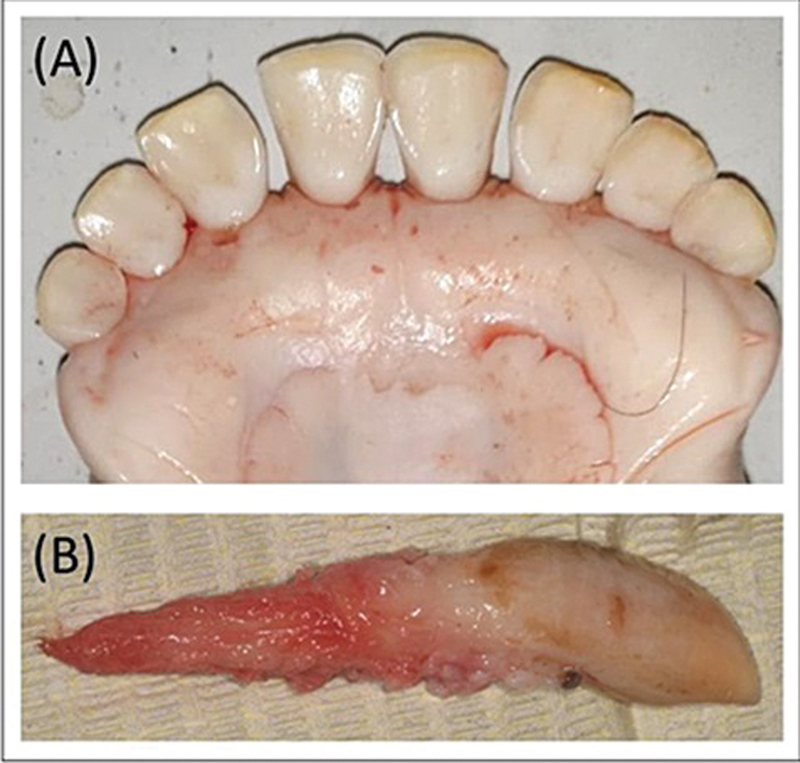
Bovine teeth and their extraction. ( **A** ) Bovine anterior mandible segment. ( **B** ) Anterior tooth after extraction.

Ethics committee approval (D-A-S-March 23) was obtained from Ajman University in accordance with institutional research regulations. Forty-eight mandibular anterior bovine teeth with straight roots were collected from 2- to 5-year-old animals slaughtered for food processing by certified providers ( Fig. 1 ). Extraction employed straight elevators for luxation followed by complete removal with upper third molar forceps, taking care to prevent fractures. After cleaning with a #11 scalpel to remove soft tissue fragments, teeth underwent immersion in 1.3% NaOCl solution for 30 seconds before thorough washing under tap water. Specimens were stored in saline at 5°C until further processing.

**Fig. 2:**
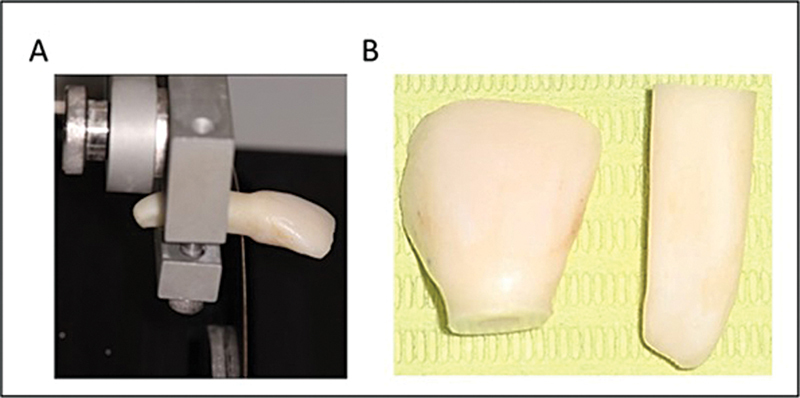
Tooth decoronation. ( **A** ) Tooth mount to the precision saw in a horizontal direction against the saw blade. ( **B** ) Tooth parts after decoronation.

**Fig. 3:**
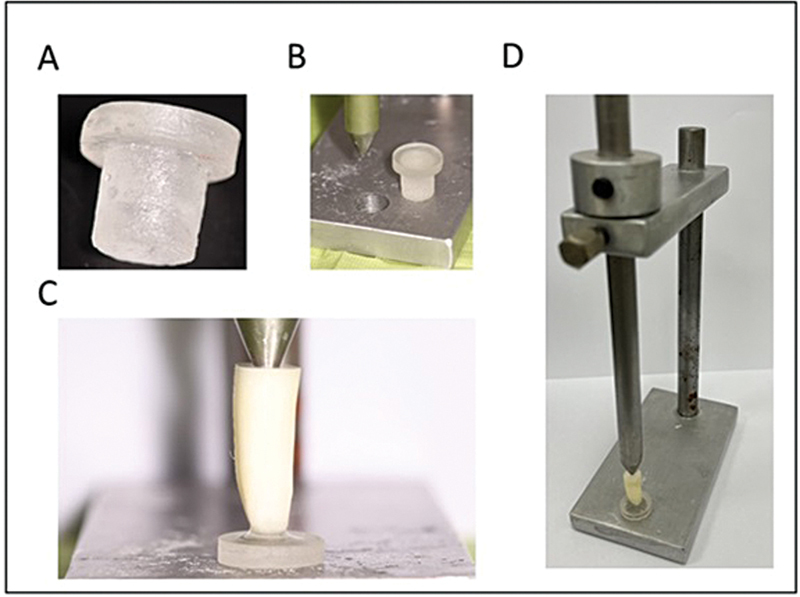
Mounting teeth roots for sectioning. ( **A** ) Acrylic stump. ( **B** ) Acrylic stump positioning in the centering device. ( **C** ) The root is positioned in a perpendicular position and held in the centering device. ( **D** ) Centering device with the root held in position to the acrylic stump.

**Fig. 4:**
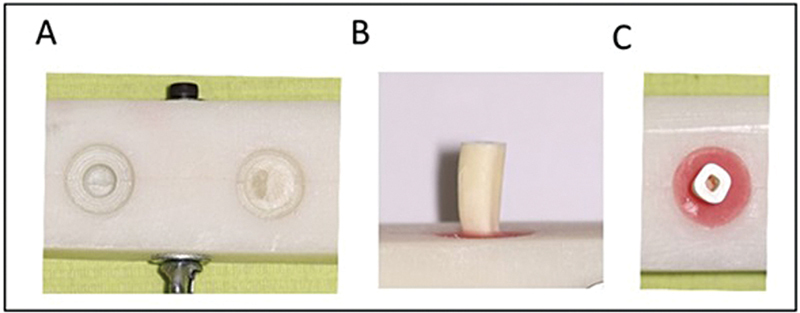
Root mounting to the sectioning base holder. ( **A** ) Custom-made mold. ( **B** ) Root mount to acrylic stump with self-cured acryl (Frontal view). ( **C** ) Root mounted to acrylic stump with self-cured acrylic (vertical view).

Two types of specimens were prepared: enamel-dentin discs for cutting efficiency assessment and cementum-dentin plates for testing intracanal lateral forces. For the splitting forces test, teeth were decoronated at the cemento-enamel junction using an IsoMet 1,000 Precision Sectioning Saw (Buehler; United Kingdom) with a 0.5 mm diamond disc operating at 275 rpm under lubricant cooling ( Fig. 2 ). Pulp tissue was removed from the crown and root using barbed broaches sized #15-40. Using a centering device, roots were mounted perpendicular to a custom-made acrylic base ( Fig. 3A–D ), enabling vertical positioning for cuts parallel to the root canal's long axis. The root tip was initially secured with Alteco Super Glue (3 g, Japan) before transferring to a custom-made mold for additional fixation with self-cured acrylic ( Fig. 4A–C ).

**Fig. 5:**
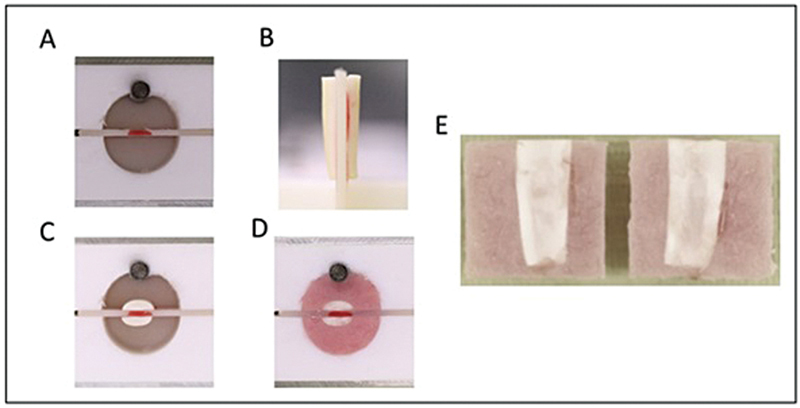
Root plates embedding for intracanal lateral forces measurement. ( **A** ) Custom-made mold with a splitting plate. ( **B** ) Root plates fixed with utility wax against each other on the splitting plate. ( **C** ) Root plates and splitting plate positioned in the Teflon mold. ( **D** ) Sample cast with self-cured acrylic. ( **E** ) End the split model after the mold disassembling.

Discarding the middle canal portion to obtain flat surfaces on the inner dentin part of the bilateral cementum-dentin plates. These root segments were embedded in an acrylic resin split model using a two-compartment cylindrical Teflon mold with a nonsticking separator ( Fig. 5A ). Root parts were positioned against the separator using utility wax ( Fig. 5B, C ), then the assembly was placed in the mold and surrounded with self-cured acrylic ( Fig. 5D ). After setting, the split model was retrieved by disassembling the mold ( Fig. 5E ).

**Fig. 6:**
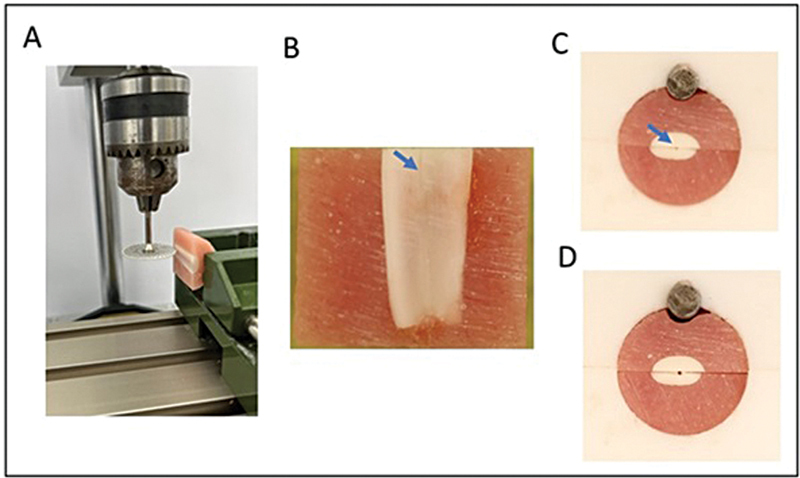
Canal customization. ( **A** ) One model part is held to a precision bench provided with a cutting disc aligned in the horizontal direction. ( **B** ) An artificial canal was milled into one of the sample parts. ( **C** ) The sample was assembled in a Teflon holder with the same dimensions as the sample. ( **D** ) Canal is further prepared to create a glide path.

The mounted sample was secured in a holder, aligning the root with the sectioning disc's vertical plane. Two parallel corono-apical cuts were made on either side of the root canal ( Fig. 6 ). A standardized testing canal measuring 0.2 mm in width and 0.2 mm in depth was created on one sample part using a precision bench with a 0.2 mm separation disc mounted horizontally on a drilling machine ( Fig. 6A ). The sample was aligned horizontally in the z-axis, creating the canal along the root's long axis ( Fig. 6B ). Sample parts were rejoined in a Teflon custom-made holder ( Fig. 6C ), and the canal was further standardized by preparing a glide path with WOG Glider (Dentsply Sirona; Johnson City, Tennessee United States; Fig. 6D ). Samples were stored in normal saline at 5°C pending testing.

**Fig. 7:**
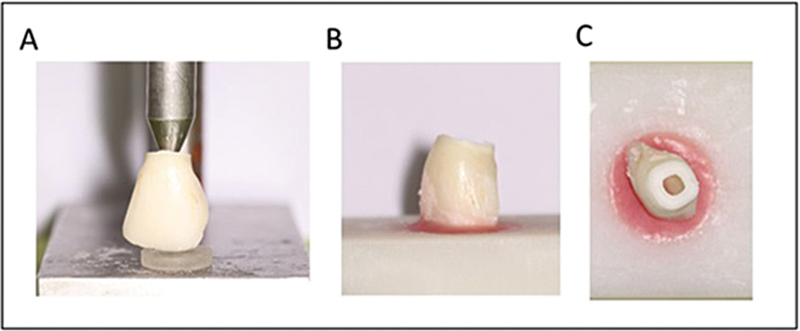
Preparing the crown for sectioning to obtain the enamel-dentin disc. ( **A** ) Tooth aligned perpendicular to acrylic stump with centering device. ( **B** ) Fixation of the crown with acryl (frontal view). ( **C** ) Fixation of the crown with acryl (vertical view).

**Fig. 8:**
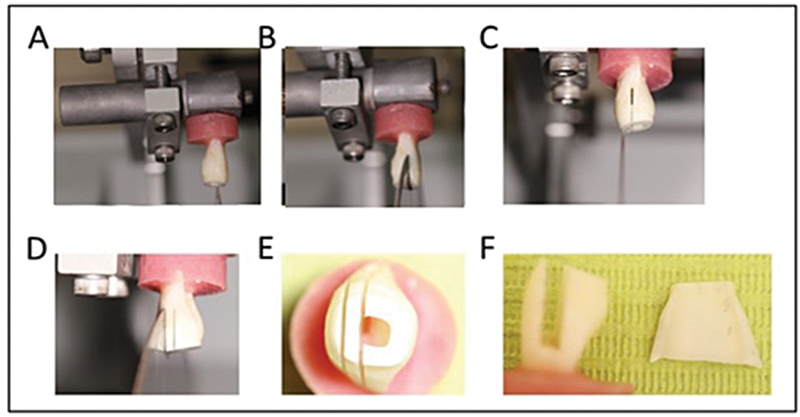
Crown sectioning to obtain an enamel-dentin disc. ( **A** ) Crown mount to the precision saw. ( **B** ) First cut made in the crown labial aspect. ( **C** ) Positioning of the saw at a 1 mm distance from the first cut. ( **D** ) The second parallel cut was made. ( **E** ) Apical view of the crown showing the cuts. ( **F** ) Enamel-dentin disc obtained.

For lateral cutting efficiency specimens, crowns were mounted perpendicular to a custom-made acrylic stump, adhered with super glue at the incisal edge, transferred to a Teflon holder, and secured with self-cured acrylic ( Fig. 7 ). The sample was mounted to the IsoMet cutting disc parallel to the crown's mesio-distal aspect, two parallel cuts spaced 1 mm apart were made in the labial aspect, yielding a 1 mm thick enamel-dentin disc ( Fig. 8 ). Each disc was divided into two pairs, one assigned to each test group, and mounted perpendicular to a holding acrylic base with the dentin facing upward. All prepared specimens were stored in normal saline at 5°C until testing.

Specimens were randomly assigned to testing groups: WOG ( *n* = 13) and Pro-Taper GOLD (PTG; *n* = 13) for lateral cutting efficiency testing, and WOG ( *n* = 24) and PTG ( *n* = 24) for splitting forces evaluation.

**Fig. 9:**
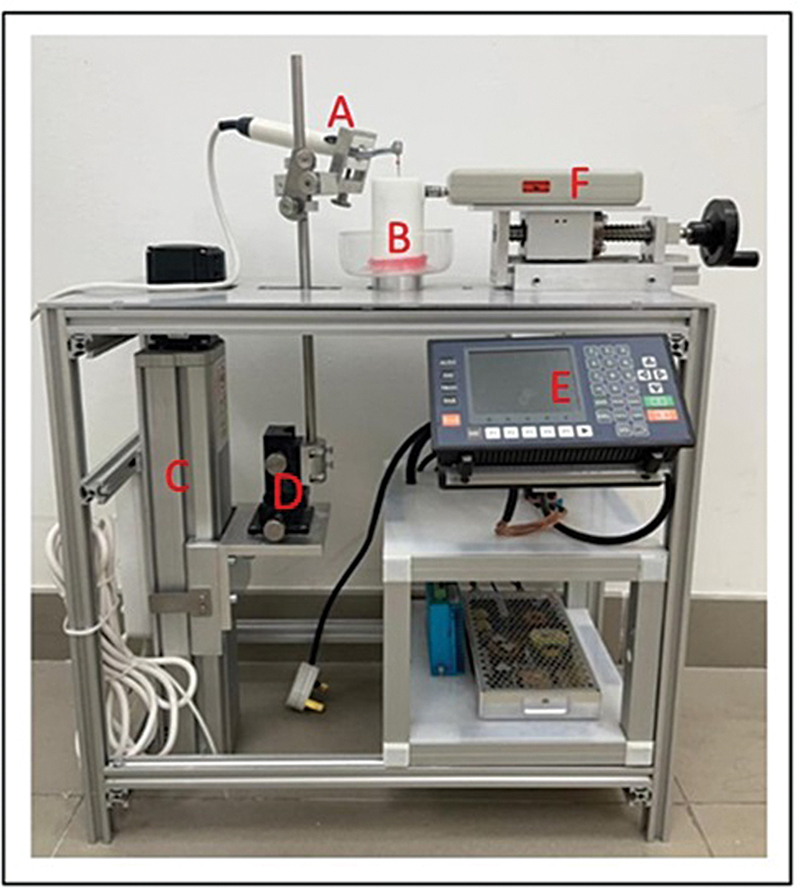
Intracanal force testing platform. ( **A** ) Endo motor handpiece holder. ( **B** ) Sample holder. ( **C** ) Electric cylinder actuator. ( **D** ) Linear slide table for fine tuning. ( **E** ) Servo controller interface. ( **F** ) Force gauge.

**Fig. 10:**
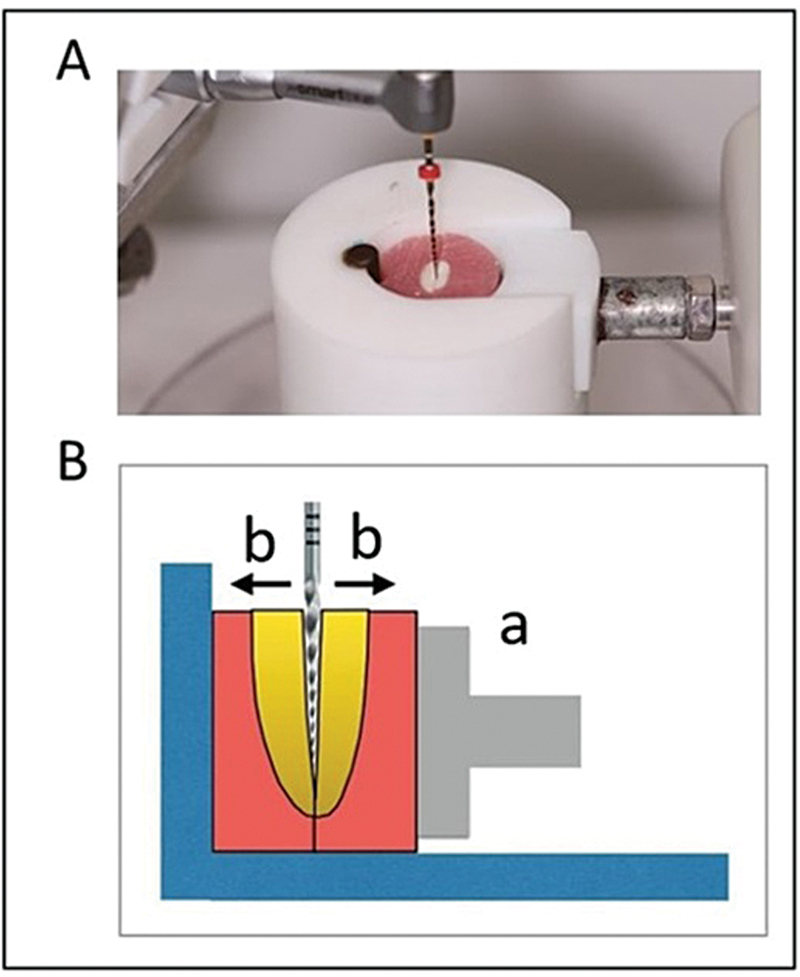
Principle of intracanal lateral force testing. ( **A** ) Actual device with file insertion in the canal. ( **B** ) Illustration of the force testing principle where ( **a** ) force gauge probe and ( **b** ) direction of splitting force.

Testing utilized a custom-built automated device, eliminating operator variability during canal preparation. The apparatus featured an endodontic motor holder, mounted to a servo motor, with samples held in a Teflon fixture, attached to a digital force gauge (DS2-50 N, ZHIQU, China, detection range: 0.1–50 N) on a linear sliding table ( Fig. 9 ). A linear sliding table mounted to the servo motor at the motor holding arm base allowed precise file-to-canal positioning. A servo controller managed the system operation. The platform detected horizontal forces generated during intracanal procedures, with pressure transmitted to the terminal force gauge ( Fig. 10 ).

**Fig. 11:**
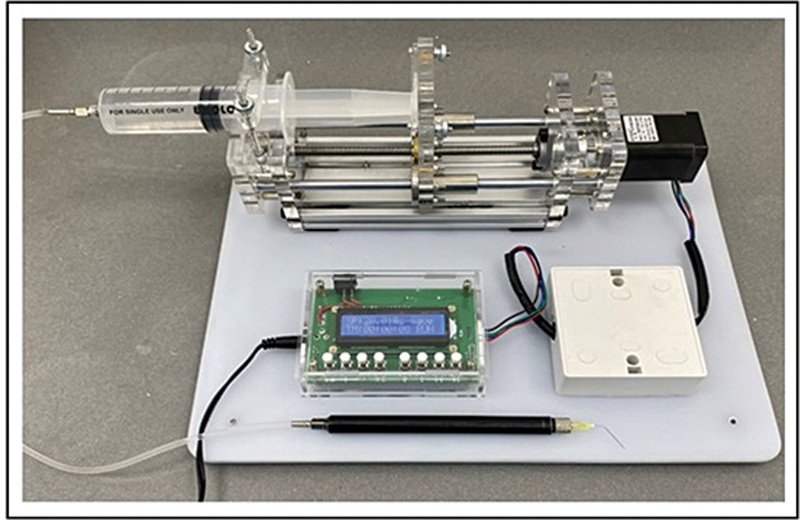
Custom-made irrigation device with diffusion control.

Canal instrumentation for splitting force testing occurred under automated conditions, with each sample using a new file or file set. Files were attached to an X-Smart Plus system (Dentsply Sirona, Switzerland) programmed according to manufacturer recommendations. For the PTG group, a complete SX-F2 file sequence was implemented, with files inserted to D16 length over three 5 mm strokes (final stroke 6 mm), except for SX, which reached D8 in one stroke. Insertion speed was set at 0.5 mm/second. After each interval, files were withdrawn, cleaned with alcohol gauze, and the canal irrigated with 1 mL of 1.3% NaOCl through a side-vented needle (ENDO-TOP, PPH CERKAMED, Poland) at 10 mL/minute using a custom irrigation device ( Fig. 11 ). For the WOG group, the Primary file was selected based on dimensional similarity to the final PTG preparation size. Instrumentation occurred in six intervals, each comprising three 1 mm picking motions, except the final increment, which advanced 1 mm in a single picking motion to reach 16 mm canal length. Between intervals, files were cleaned and canals irrigated following the same protocol as the PTG group. Force measurements were recorded at five readings per second through the manufacturer's computer interface (ZHIQU, China).

**Fig. 12:**
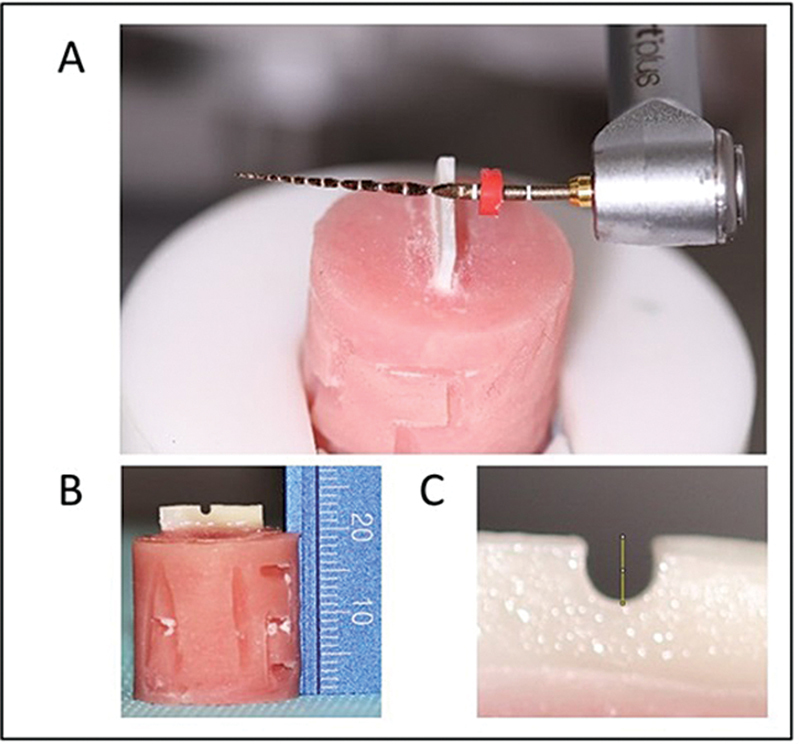
Cutting efficiency test. ( **A** ) File at D16 positioned at the dentin disc. ( **B** ) The sample after cutting efficiency test with the scale in its position. ( **C** ) Photos analysis with Image J software.

Lateral cutting efficiency testing employed an Endo-motor handpiece mounted to a free-falling holder on a ball bearing sliding cylinder, permitting vertical movement only. The holder is connected to a horizontal linear sliding system for positioning the file at D16 on the dentin disc. Vertical force resulted from the handpiece weight (186 g per manufacturer specifications plus 34 g holder weight, totaling 220 g). Files were applied perpendicular to the dentin disc for 3 minutes in both groups using the recommended X-Smart Plus system program. Continuous saline irrigation (10 mL/minute) was delivered at the file-dentin contact point. After testing, a DSLR camera photographed the sample alongside a measuring scale reference. Cutting depth was analyzed using ImageJ software (NIH and LOCI, University of Wisconsin, Madison, Wisconsin, United States), measuring from the dentin flat edge to the maximum cut point ( Fig. 12 ).

Data analysis involved identifying five peaks in each dataset, corresponding to ICSF generated during instrumentation. The highest value in each peak represented the maximum force produced. These values and cutting depths were tabulated for statistical processing using SPSS version 27.0 (IBM, Armonk, New York, United States). Normality was confirmed via Kolmogorov–Smirnov testing. Pearson's correlation evaluated relationships between splitting force and cutting efficiency. Comparisons between different files or strokes within systems employed one-way ANOVA with post hoc Tukey HSD tests, while cutting efficiency between systems was compared using one-way ANOVA.

## Results

### Intracanal Splitting Forces Results

The investigation examined ICSF generated by endodontic files in reciprocating motion (WOG) and rotary motion (PTG), measured in newtons, alongside cutting efficiency measured in millimeters. The PTG group, particularly the S1 file, produced significantly higher splitting forces (mean: 20.10 ± 5.85 N) compared to the lowest detected mean in the WOG first stroke (0.32 ± 0.41 N).

ICSF values for both systems appear in Tables 1 and 2 , with means and standard deviations presented in Table 3 . Forces generated by WOG demonstrated an ascending pattern as instrumentation progressed apically, with the first stroke producing minimal force (0.32 ± 0.41 N) and the final stroke generating the highest values (13.58 ± 3.64 N). In the PTG group, the SX file produced the lowest forces (4.24 ± 2.22 N), while the S1 file generated the highest (20.10 ± 5.85 N).

**Table 1:** ICSF of each sample in the WOG group measured in newtons (N)

Sample no.	Stroke 1	Stroke 2	Stroke 3	Stroke 4	Stroke 5	Stroke 6
1	0.1	0.41	0.83	6.61	9.2	10.1
2	0.41	0.02	2.35	7.26	10.71	10.67
3	0.05	0.04	0.09	5.59	8.98	8.92
4	0.23	3.3	0.1	6.05	8.83	9.94
5	0.07	0.03	1.55	7.16	9.64	9.93
6	0.05	0.06	0.41	8.17	10.93	10.4
7	1.49	6.4	8.79	11.44	13.66	14.37
8	0.03	0.02	1.23	4.72	8.19	8.4
9	0.37	3.56	8.99	10.71	12.71	12.48
10	0.05	0.99	6.53	12.84	14.73	14.84
11	0.09	2.73	8.15	9.33	15.76	15.5
12	0.14	4.21	5.21	6.48	6.93	11.02
13	0.16	3.4	10.94	7.82	18.65	19.02
14	0.05	3.26	6.38	7.51	11.13	11.79
15	0.97	5.45	9.5	10.4	11.65	11.24
16	0.25	6.03	8.43	10.55	8.79	10.9
17	1.02	9.55	8.52	9.43	10.48	14.36
18	0.95	0.87	6.37	7.5	8.1	16.9
19	0.08	0.75	3.59	5.38	7.45	15.23
20	0.82	2.93	9.1	8.76	7.16	20.74
21	0.15	3.86	5.15	10.23	9.9	14.57
22	0.09	1.05	5.45	10.48	4.69	20.58
23	0.5	1.82	6.76	7.55	6.25	12.28
24	0.36	1.41	6.88	11.08	13.54	18.16

**Table 2:** ICSF of each sample in the PTG group measured in newtons (N)

Sample no.	SX	S1	S2	F1	F2
1	1.69	15.72	8.46	5.21	7.37
2	3.12	9.16	2.79	1.96	6.47
3	5.83	10.31	6.15	4.65	5.18
4	1.4	16.01	11.18	4.94	6.05
5	6.15	15.96	14.03	7.72	10.99
6	5.15	17.2	9.02	3.3	3.71
7	7.62	23.01	14.08	7.41	9.86
8	4.02	19.35	8.79	8.96	9.4
9	3.43	9.69	8.79	5.79	6.69
10	8.17	23.93	21.65	11.69	15.12
11	3.77	23.39	12.08	11.75	10.31
12	1.61	14.61	5.25	7.77	8.62
13	3.47	20.07	6.18	6.77	9.48
14	2.66	18.58	5.54	5.32	10.16
15	3.49	29.5	7.3	5.45	4.94
16	7.21	24.21	16.36	13.26	10.52
17	2.97	22.84	9.5	9.03	3.46
18	2.39	31.53	24.89	15.39	25.8
19	8.35	26.06	23.13	16.28	16.62
20	1.25	21.41	12.89	17.48	12.71
21	5.84	23.7	10.37	20.28	4.83
22	2.6	24.21	20.15	17.35	13.8
23	2.97	24.28	15.6	11.26	16.83
24	6,051	17.74	14.67	10.22	8.12

**Table 3:** Mean values and standard deviations of the test groups presented for each file/stroke (N)

WaveOne Gold			Pro-Taper Gold		
Stroke	Mean (N)	SD	File	Mean (N)	SD
1	0.32	0.41	SX	4.24	2.22
2	2.59	2.47	S1	20.10	5.85
3	5.47	3.40	S2	12.04	5.94
4	8.46	2.17	F1	9.55	5.00
5	10.34	3.26	F2	9.90	5.22
6	13.58	3.64			

### Statistical Comparisons

Statistical analysis via one-way ANOVA revealed significant differences between test groups ( *p* < 0.001; Table 4 ). Post hoc Tukey HSD analysis of the WOG group identified significant differences between stroke 1 and strokes 3, 4, 5, and 6; between stroke 2 and strokes 4, 5, and 6; and between strokes 4 and 6. No significant differences emerged between other stroke comparisons ( Table 5 ). For the PTG group, significant differences appeared between the S1 file and all other files, and between the S2 and SX files, while no significant differences emerged among other file comparisons ( Table 6 ).

**Table 4:** ANOVA statistics for ICSF between test groups

ANOVA					
Splitting forces (N)					
	Sum of squares	df	Mean square	F	Sig.
Between groups	530.005	1	530.005	22.352	<0.001
Within groups	1,090.760	46	23.712		
Total	1,620.765	47			

**Table 5:** Multiple comparison among WOG group (post hoc Tukey HSD)

Stroke	Other strokes	Mean difference	*p* -Value
1	2	−2.266250	0.059
	3	−5.147500	<0.001
	4	−8.135833	<0.001
	5	−10.012500	<0.001
	6	−13.252319	<0.001
2	1	2.266250	0.059
	3	−2.881250	0.006
	4	−5.869583	<0.001
	5	−7.746250	<0.001
	6	−10.986069	<0.001
3	1	5.147500	<0.001
	2	2.881250	0.006
	4	−2.988333	0.004
	5	−4.865000	<0.001
	6	−8.104819	<0.001
4	1	8.135833	<0.001
	2	5.869583	<0.001
	3	2.988333	0.004
	5	−1.876667	0.184
	6	−5.116486	<0.001
5	1	10.012500	<0.001
	2	7.746250	<0.001
	3	4.865000	<0.001
	4	1.876667	0.184
	6	−3.239819	0.001
6	1	13.252319	<0.001
	2	10.986069	<0.001
	3	8.104819	<0.001
	4	5.116486	<0.001
	5	3.239819	0.001

**Table 6:** Multiple comparison among the PTG group (post hoc Tukey HSD)

	Other files	Mean difference	*p* -Value
SX	S1	−15.866667	<0.001
	S2	−7.799167	<0.001
	F1	−5.315417	0.003
	F2	−5.661141	0.002
S1	SX	15.866667	<0.001
	S2	8.067500	<0.001
	F1	10.551250	<0.001
	F2	10.205525	<0.001
S2	SX	7.799167	<0.001
	S1	−8.067500	<0.001
	F1	2.483750	0.431
	F2	2.138025	0.592
F1	SX	5.315417	0.003
	S1	−10.551250	<0.001
	S2	−2.483750	0.431
	F2	−0.345725	0.999
F2	SX	5.661141	0.002
	S1	−10.205525	<0.001
	S2	−2.138025	0.592
	F1	0.345725	0.999

### Cutting Efficiency Results

The cutting efficiency measurements, presented in Table 7 , demonstrated superior performance in the WOG group compared to the PTG group (1.52 ± 0.41 and 1.08 ± 0.43 mm, respectively). The WOG group exhibited boundaries from 1.05 to 2.09 mm, while the PTG group ranged from 0.46 to 1.9 mm. One-way ANOVA confirmed a significant difference between the experimental groups ( *p* = 0.014; Table 8 ).

**Table 7:** Cutting efficiency of WOG and PTG groups measured in mm

Sample number	Group A (WaveOne Gold); primary file	Group B (ProTaper-Gold); F2 file
1	1.07	1.47
2	2.04	1.04
3	1.39	0.85
4	2.09	1.90
5	2.2	1.86
6	1.43	1.29
7	1.54	0.81
8	1.90	1.07
9	1.17	0.94
10	1.11	0.46
11	1.32	0.73
12	1.43	0.85
13	1.05	0.83
Mean	1.52	1.08
SD	0.41	0.43

**Table 8:** One-way ANOVA statistical analysis for inter-group differences in cutting efficiency

ANOVA					
Cutting efficiency (mm)					
	Sum of squares	df	Mean square	F	Sig.
Between groups	1.228	1	1.228	6.950	0.014
Within groups	4.241	24	0.177		
Total	5.470	25			

### Correlation Analysis

However, Pearson correlation testing found no significant relationship between splitting force and cutting efficiency for either testing group ( Tables 9 and 10 ).

**Table 9:** Pearson correlation test between ICSF and cutting efficiency in the WOG group

		Splitting forces (N) max.—WaveOne-Gold	Cutting efficiency (mm)—WaveOne-Gold
Splitting forces (N) max.—WaveOne-Gold	Pearson correlation	1	−0.530
	Sig. (two-tailed)		0.062
	N	24	13
Cutting efficiency (mm)—WaveOne-Gold	Pearson correlation	−0.530	1
	Sig. (two-tailed)	0.062	
	N	13	13

**Table 10:** Pearson correlation test between ICSF and cutting efficiency in the PTG group

		Splitting forces (N) max.—ProTaper-Gold	Cutting efficiency (mm)—ProTaper-Gold
Splitting forces (N) max.—ProTaper-Gold	Pearson correlation	1	−0.291
	Sig. (two-tailed)		0.335
	N	24	13
Cutting efficiency (mm)—ProTaper-Gold	Pearson Correlation	−0.291	1
	Sig. (two-tailed)	0.335	
	N	13	13

## Discussion

File selection was based on achieving equivalent final preparation dimensions. The WOG Primary file (tip size: 25, variable taper: 7–3%) and PTG F2 file (tip size: 25, 8% taper) were chosen as they produce close final canal dimensions at the working length. Both systems used the WOG Glider for standardized glide path creation, ensuring comparable starting conditions.

This study confirmed the reciprocating WOG system's significantly superior lateral cutting efficiency compared to the rotary PTG system. This enhanced efficiency, however, did not influence ICSF during simulated premolar instrumentation. Based on this finding null hypothesis was rejected, despite no intergroup ICSF differences. In addition, cutting efficiency showed no correlation with ICSF.

In the PTG system, the S1 file generated the highest ICSF (20.10 ± 5.85 N), exceeding Deari et al's findings (15.8 ± 4.5 N) and all WOG strokes. 28 This also surpasses the approximately 14 N safety threshold established by Holcomb et al. 29 Despite its larger taper (3.5–19% between D1 and D9) and diameter (1.2 mm at D14), the SX file showed the lowest ICSF among PTG files, likely due to shallower insertion depth. 30 31

Higher ICSF in PTG may stem from extended inward sliding motions. Gambarini et al noted that inward pecking with mild apical pressure creates greater torque than upward brushing. 32

WOG's peak ICSFs occurred during apical preparation (strokes 5 and 6: 10.34 ± 3.26; 13.58 ± 3.64), while PTG's highest values emerged during coronal and middle third shaping (S1 and S2: 20.10 ± 5.85; 12.04 ± 5.94). This difference may relate to working length file diameters: WOG Primary terminated at D16 (1.135 mm), while PTG's S1 reached 1.2 mm at D16. 30 Both systems used the WOG Glider (D16 = 0.85 mm) for glide path creation.

The larger diameter disparity between the WOG glider and the S1 file, alongside PTG's longer inward motion, likely contributed to elevated ICSF. This aligns with Blum et al, who showed coronal shaping files contact 3 to 7 mm from nonactive tips, generating maximum torque. 33 As Thu et al suggested, increasing taper enhances surface area and contact. 34 WOG's lower ICSF may relate to reduced insertion depth and progressive pecking motion, except for the final stroke. Though root taper's role in fracture risk remains unclear, 35 excessive enlargement may compromise root strength. 29

Cutting efficiency showed no significant correlation with ICSF, echoing Deari et al. 28 The WOG Primary file demonstrated higher cutting efficiency than PTG F2, possibly due to differences in flute number, cross-section, tip design, and blade configuration. 36 Despite identical geometry, PTG F2 showed greater efficiency than PTU F2, 28 potentially due to metallurgy and heat treatment. 37 38 WOG removed less dentin volume than PTG, 39 consistent with results from S-shaped canals favoring PTG for coronal shaping. 40 Poor debris removal may impair cutting and increase torque and crack formation, 36 41 though ICSF remained unaffected in this study despite reportedly more debris accumulation occurring with reciprocating systems. 42

To prevent performance degradation, files were used once per sample. 43 *In vitro* , WOG produced more dentinal defects than PTG across all canal thirds, 44 likely reflecting differences in cutting efficiency rather than force. Sequential file use reduces contact areas, improves cutting, and lowers torque. 33 Crown-down techniques produce lower torque than step-back or single-file approaches, 45 though initial coronal files in crown-down techniques induce more stress. 33 Thu et al confirmed deeper file penetration correlates with increased torque, potentially explaining higher ICSF in PTG (5–6 mm strokes) compared to WOG (1 mm pecking). 38

The 1.3% NaOCl concentration used in this study represents a clinically relevant irrigation protocol that provides effective antimicrobial action while minimizing the risk of excessive tissue dissolution that could affect the mechanical properties of the test specimens. 46 Zhang et al found that 1.3% NaOCl caused insignificant changes in collagen degradation and flexural strength when used for up to 4 hours, unlike 5.25% NaOCl, which significantly reduced these properties after 1 hour.

Specimens were stored in normal saline at 5°C for less than 30 days before testing. This storage protocol has been validated in previous studies and maintains dentin mechanical properties without significant degradation. Saline storage substantially reduces hardness after 30 days, though other mechanical properties remain relatively stable. 47 On the other hand, it preserves the hydration state of dentin, which is crucial for maintaining realistic mechanical behavior during force testing.

Repeated glide-path instrumentation reduces torque in subsequent files. 48 49 This factor was standardized in this study by using the WOG Glider (D0 = 0.15 mm, taper 2%; D16 = 0.85 mm, taper 6%) in both groups. Kwak et al demonstrated that glide path and preflaring significantly reduce torque during MB2 shaping. 50

File cross-section impacts torque; convex triangular files (PTG) generate more torque than parallelograms (WOG). 51 Lubricants also influence torque—aqueous solutions like NaOCl reduce force more effectively than pastes 52 and are recommended with WOG. 53

It seems that file design significantly impacts ICSF and canal stress, influencing fracture risk. 54 55 WOG contacts the canal with only one to two edges, reducing torque while improving debris removal. 56 On the other hand, PTG has a neutral rake angle with passive cutting. 57 Active cutting files produce less wedging than passive ones, 36 and smaller tapers with enhanced cutting reduce craze lines. 18

Manufacturers claim WOG's reciprocating motion reduces screw-in force, with clockwise rotation relieving entrapment during counter-clockwise movement. Though motion type matters, heat treatment and geometry exert greater influence. 51 Square cross-sections cause maximum screw-in force, followed by rectangular and triangular; slender rectangular (WOG) produced the least. 58 In our study, we controlled screw-in force using a linear actuator (0.5 mm/second insertion rate).

Despite existing research, the relationship between NiTi file design and VRF remains underexplored. Kim et al reported that greater taper and single-file systems reduce fracture resistance. 54 No consensus exists on clinically superior file designs, and long-term outcomes related to engine-driven systems are lacking. 49

A major strength of this study is the force application standardization via testing apparatus, eliminating operator variability. 27 59 However, the precisely applied force remains unknown. Standardization of canal shape, pressure, and motion enhances inter-group comparison, unlike clinical variability.

Research on downward load effects on NiTi performance is sparse. 50 Crack formation has often been overestimated due to flawed methods. 60 No RCTs have evaluated whether lower-taper files reduce fracture risk, and prospective studies are impractical due to lengthy follow-ups and multifactorial influences. FEA could provide standardized insight into force distribution across root anatomies. 35

Limitations of this investigation, in addition to being an *in vitro* study, are that the lateral cutting efficiency findings may not translate clinically, as debris removal varies with file design. 42 The periodontal ligament was also excluded to isolate intracanal forces, which eliminates tactile feedback but avoids damping effects. The automated setup ensures consistent motion but lacks real-time adaptation.

Considering curved canals, existing evidence indicates that reciprocating systems tend to maintain original canal curvature more effectively and reduce torsional stress compared with continuous rotary systems. 61 In line with our findings of lower ICSF, the WOG system may, therefore, be advantageous for shaping curved canals while minimizing stress concentration and preserving canal anatomy. By contrast, the multi-file, continuous rotation sequence of PTG allows progressive enlargement and effective shaping but may produce higher stresses in curved or complex canal systems because of repeated file insertions and extended inward movements. Clinicians should weigh these differences when selecting instruments for curved root canals, balancing the need for efficient shaping against the potential for increased dentinal stress.

## Conclusion

- Lateral cutting efficiency in the rotary system (PTG) was significantly higher than the reciprocating system (WOG) yet showed no correlation to generated ICSF.
- Despite identical heat treatment, ICSF in PTG was significantly higher than WOG, suggesting file geometries (especially cross-section), kinematics, stroke parameters, and maximum coronal diameters substantially influence ICSF generation.
- In PTG, shaping files (S1, S2) produced the highest ICSF among both groups.
- Precoronal flaring with SX appears ineffective at reducing ICSF; increased stroke numbers and pecking motions might be more beneficial.
- In WOG, the lowest ICSF values occurred in coronal/middle thirds, while the highest values emerged as the file progressed apically.
- Further investigation is needed regarding the ICSF/VRF relationship with torque generation. And additional research on precise factors causing the wedging effect/ICSF is necessary due to the limited existing studies.

### Clinical Significance

From a clinical decision-making standpoint, the markedly lower intracanal splitting force (ICSF) values observed with the WOG system indicate it may be a safer option in situations where the risk of VRF is heightened—such as teeth with thin root walls, previous endodontic treatment, or extensive coronal destruction. Conversely, the greater cutting efficiency demonstrated by PTG may offer a distinct advantage in highly calcified or sclerotic canals where more aggressive dentin removal is required to achieve adequate shaping. Therefore, clinicians should balance the reduced fracture risk of WOG against the enhanced shaping efficiency of PTG and select the system that best matches the anatomical and structural characteristics of each case.
